# Innate Immune Cells in the Esophageal Tumor Microenvironment

**DOI:** 10.3389/fimmu.2021.654731

**Published:** 2021-04-28

**Authors:** Kele Cui, Shouxin Hu, Xinyu Mei, Min Cheng

**Affiliations:** ^1^ Department of Clinical Laboratory, The First Affiliated Hospital of USTC, Division of Life Sciences and Medicine, University of Science and Technology of China, Hefei, China; ^2^ Department of Geriatrics, Gerontology Institute of Anhui Province, The First Affiliated Hospital of USTC, Division of Life Sciences and Medicine, University of Science and Technology of China, Hefei, China; ^3^ Anhui Provincial Key Laboratory of Tumor Immunotherapy and Nutrition Therapy, Hefei, China; ^4^ Cancer Immunotherapy Center, The First Affiliated Hospital of USTC, Division of Life Sciences and Medicine, University of Science and Technology of China, Hefei, China; ^5^ Department of Thoracic Surgery, The First Affiliated Hospital of USTC, Division of Life Sciences and Medicine, University of Science and Technology of China, Hefei, China

**Keywords:** innate immune cells, crosstalk, regulation, esophageal tumor microenvironment, immunotherapy strategy

## Abstract

Esophageal cancer (EC) is one of the most common mucosa-associated tumors, and is characterized by aggressiveness, poor prognosis, and unfavorable patient survival rates. As an organ directly exposed to the risk of foodborne infection, the esophageal mucosa harbors distinct populations of innate immune cells, which play vital roles in both maintenance of esophageal homeostasis and immune defense and surveillance during mucosal anti-infection and anti-tumor responses. In this review, we highlight recent progress in research into innate immune cells in the microenvironment of EC, including lymphatic lineages, such as natural killer and γδT cells, and myeloid lineages, including macrophages, dendritic cells, neutrophils, myeloid-derived suppressor cells, mast cells and eosinophils. Further, putative innate immune cellular and molecular mechanisms involved in tumor occurrence and progression are discussed, to highlight potential directions for the development of new biomarkers and effective intervention targets, which can hopefully be applied in long-term multilevel clinical EC treatment. Fully understanding the innate immunological mechanisms involved in esophageal mucosa carcinogenesis is of great significance for clinical immunotherapy and prognosis prediction for patients with EC.

## Introduction

According to an analysis of 36 cancer types in 185 countries, esophageal cancer (EC) accounts for approximately 3.2% of incidence and 5.3% of mortality attributable to total cancers (1). Risk factors for EC include smoking, alcohol consumption, low fruit intake, and high body-mass index, and it is becoming a major disease burden worldwide (2). There are two main types of EC, esophageal adenocarcinoma (EAC) and esophageal squamous cell carcinoma (ESCC) (3). EAC is most common in developed countries (e.g., Europe and America), while ESCC mainly occurs in developing countries, including eastern Asia and Africa, and particularly China, which had the highest age-standardized incidence, mortality, and disability-adjusted life-years rates among 195 countries in 2017 (2). Due to the lack of reliable diagnostic indicators, the prognosis of patients with EC remains relatively poor; while surgical resection can prolong patient survival, rates of recurrence and metastasis remain high, with 5-year survival rates only 15%–25% (3, 4). In many patients who cannot benefit from esophagectomy, immune status determines sensitivity to radiotherapy and chemotherapy (3).

The recent realization that the involvement of innate immune system in the process of defending mucosal-associated infection and tumors has fuelled the accelerated interest in the roles of innate immune cells in the pathogenesis of EC. The esophageal mucosa harbors numerous innate immune cells, which is attributed to their quick responses when encountering foreign foodborne antigens (4, 5). The partial exposure of the esophageal mucosa to the external environment makes it vulnerable to pathogen attack, which can cause long-term inflammation that may develop into esophageal dysplasia and subsequently cancer (6). Via recognizing molecular alterations caused by microbial infections (7) or cancer cells with multiple genetic mutations (8), innate immune cells can induce effector responses such as cytotoxicity by natural killer (NK) cells and phagocytosis by macrophages. Besides, they can initiate adaptive immune responses by antigen presentation to tumor-specific CD8^+^ T antigen-presenting cells (APCs) (9). Innate immune cells can also exert effector functions after antibody induction, including antibody-dependent cellular cytotoxicity or phagocytosis, which rely on Fc receptor (FcR) expression (9). Cancer cells can escape from anti-tumor immune responses by promoting polarization toward immunosuppressive cell phenotypes, including tumor-associated macrophages (TAMs) and dendritic cells (DCs), recruiting immunomodulatory cells, such as myeloid-derived suppressor cells (MDSCs) and regulatory T cells (Tregs), and inducing over-expression of immune checkpoint molecules by NK or T cells (10). Due to the plasticity of innate immune cells, another strategy of tumor immune escape is to enable them to orchestrate the angiogenic switch under tumor microenvironment (TME) stimuli, to support the tumor progression (5, 11). Given the important defensive and regulatory roles of innate immune cells in cancer progression, extensive attention has been focused on their pathogenic or protective functions in the microenvironment of many solid tumors (12). Clinical immunotherapy approaches based on innate immune cells, including inhibitors targeting immune checkpoints, such as PD1/PD-L1, CTLA4, TIGIT, CD96, TIM3, and LAG3, as well as bispecific antibodies or chimeric antigen receptor (CAR) T cells, to promote specific T cell responses, have been extensively studies and have potential for use as adjuvant therapies, alongside surgical resection and chemoradiotherapy, to treat cancers (9, 13). Therefore, adequate understanding of how variations of innate immunity in the TME affect EC pathogenesis is of great practical significance for clinical treatment. However, until now, the roles of innate immune cells in EC have not been comprehensively described.

Herein, we review recent progress in understanding of the roles of innate immune cells, including NK cells, γδT cells, TAMs, DCs, MDSCs, neutrophils, mast cells (MCs) and eosinophils in the TME of EC, as well as the underlying cellular and molecular mechanisms involved in tumor occurrence and progression, with the aim of providing directions for combined immunotherapy strategies and prognosis prediction.

## NK Cells

**Figure 1:**
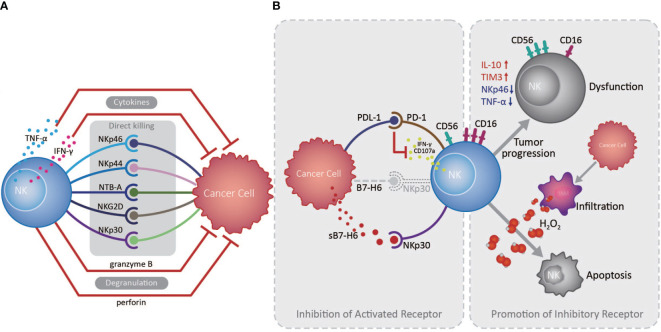
Roles of natural killer cells in the esophageal cancer tumor microenvironment. **(A)** Natural killer (NK) cells exert essential anti-tumor functions through degranulation, cytokine release, and activated receptor expression, directly killing esophageal tumor cells in the absence of antigen recognition in the tumor microenvironment (TME). **(B)** Cancer cells can escape from NK cell immune surveillance in the TME through the PD-1/PD-L1 pathway and the competitive combination of activated NKp30 receptor, expressed on NK cells, by releasing soluble B7-H6 (indicated as sB7-H6). Otherwise, large amounts of H_2_O_2_ produced by tumor infiltrated macrophages in the TME are prone to induce CD56^dim^ NK cell apoptosis, while CD56^dim^ NK cells can also transform to a CD56^bright^ phenotype during tumor development, as well as decreasing in number and becoming dysfunctional.

The innate immune system serves as the front line of host defense against pathogen invasion and tumor, in which NK cells play a vanguard role due to their powerful cytotoxic activity (14). NK cells express various activating and inhibitory receptors for tumor cell recognition and are the primary force in innate anti-tumor immune surveillance, playing vital roles in inhibiting cancer development at early stages, and in controlling cancer metastasis (13, 15). NK cells can initiate anti-tumor responses through directly killing tumor cells, secreting cytokines, including IFN-γ and TNF-α, and recruiting other anti-tumor immune cells (13). Studies indicated that infiltrating NK cell density in the EC TME is positively correlated with patient overall survival (OS) and favorable postoperative prognosis (16, 17). NK cells in the TME of EC can recognize and kill tumor cells *via* NKp30/B7-H6 pathway, which substantially contributes to NK cell-mediated immune responses (16). However, EC patients with high B7-H6 expression have inferior survival, primarily because EC cells can secret soluble B7-H6, which competitively binds the NKp30 receptor on NK cells, thereby inhibiting NKp30-mediated killing (16). Interestingly, IL-17 secreted by CD4^+^Foxp3^-^ Th17 cells can increase NK cell numbers by stimulating EC cells to produce chemokines (CXCL9 and CXCL10), and augment NK cell activation and function by enhancing their TNF-α, IFN-γ, granzyme B, and perforin production, and the expression of activating receptors (NKp46, NKp44, NTB-A, and NKG2D) (17, 18). Nevertheless, an important mechanism by which tumor cells counteract NK cells is by promoting over-expression of immune checkpoints and down-regulation of activating receptors on NK cells, inducing their dysfunction and exhaustion (13). Up-regulated expression of the inhibitory receptor, Tim-3, on EC tumor-infiltrated NK cells is accompanied by NK cell dysfunction and exhaustion, and associated with tumor invasion depth, nodal status, and advanced clinical stage (19). Furthermore, increased PD1 expression on peripheral and tumor-infiltrating NK cells can inhibit their IFN-γ secretion and CD107a expression through the PD-1/PD-L1 pathway, and is associated with poor survival of EC patients (20) (**Figure 1**).

Cancer cells can promote a suppressive TME, which challenges anti-tumor immunity by inducing an imbalance in activating and inhibitory immune cell signaling, suppressive factor secretion, and recruitment of suppressive immune cells. In principle, CD56^dim^ NK cells, which exhibit higher cytotoxicity, are more sensitive to apoptosis than CD56^bright^ NK cells in the presence of physiological H_2_O_2_ levels; large amounts of H_2_O_2_ can be produced in the TME of EC, which contributes to reduced infiltration by CD56^dim^ NK cells as tumors develop (21). Consistently, NK cell numbers in the circulation and omentum of patients with EAC were significantly reduced and skewed toward the CD56^bright^ phenotype with increased IL-10 and reduced NKp46 and TNF-α production, exhibiting reduced toxicity and inhibited function (22). Similarly, in ESCC patients, with the down-regulation of CD16 and increased expression of CD56, the NK cell levels were also declined in the tumors and exhibited an exhausted phenotype (23, 24), demonstrating the vital roles of the suppressive microenvironment formed by cancer cells in altering NK cell phenotype and activity (**Figure 1**).

Although existing studies on EC have provided a preliminary understanding of anti-tumor effects of NK cells, there is still not enough to fully outline the roles and prognostic significance of NK cells involved in EC. Recently, a study in human hepatocellular carcinoma reported that breaking the balance between active receptor CD226 and inhibitory receptors CD96 and TIGIT lead to impaired NK cell function (25). Other than the attenuation of targeting and killing of tumor cells and acquisition of tolerogenic/immunosuppressive behavior, tumor-associated NK cells can acquire pro-angiogenic activities favoring tumor progression due to TME stimuli in various solid malignancies including prostate cancer, lung cancer and colon cancer, which inspires us to pay attention to the full-scale immune function of NK cells in EC (11, 26). The significance of investigations on the function of NK cells lies in facilitating the development of NK cell-based immunotherapy in EC. Indeed, studies from an ESCC animal model revealed that IL-18 deficiency can down-regulate local NK cell anti-tumor immunity by decreasing their IFN-γ production, suggesting that exogenous IL-18 supplementation has potential to delay EC development (27). Furthermore, NK cells expanded *in vitro* have high cytotoxicity against ESCC cells expressing NKG2DLs, particularly those exhibiting an epithelial-mesenchymal transition (EMT) phenotype, raising the possibility of clinical therapy targeting these NK cells in patients with ESCC (18). However, much remains to be done before these findings can be applied to the clinical treatment of EC.

## γδT Cells

As important contributors to innate immunity, γδT cells perform complex roles, including immune surveillance, immune regulation, and effector functions (28); they can be divided into two types according to their T cell receptor δ chain: Vδ1 and Vδ2 T cells. The former exists in healthy epithelium and participates in maintaining epithelial homeostasis, whereas the latter is present on 70% of total peripheral γδT cells (29). Flow cytometry analysis of γδT cells from patients with EC revealed that the majority of peripheral circulatory γδT cells expressed the Vγ9 and Vδ2 T cell receptors and exhibited cytotoxicity against EC cells, mainly by recognizing heat shock protein (HSP) 60 and HSP70 on the tumor cell surface (30). However, Vδ1^+^ T cells are dominant in the TME of ESCC, possibly because activated Vδ1^+^ γδT cells in peripheral blood can adhere to ESCC cells and fibroblasts *via* adhesion molecules, including LFA-1 (CD11a), CD49d, CD49e, L-selectin, and CD103, whereas Vδ2 T cells can only use a few adhesion molecules, including LFA-1, L-selectin, and CD44v6 (31).

The role of γδT cells in EC is far from well understood. Although γδT cells make up a small population of tissue-resident lymphocytes, they constitute an important first line of defense against infections, autoimmune diseases and tumors, especially in the mucosal barrier such as the skin, lung, liver, tongue, genital tract and peritoneal cavity (32–34). However, the alterations in γδT cell subsets and functions, as well as their prognostic and diagnostic significance in the EC remains obscure. Indeed, we have been focusing on the role of tissue-resident γδT cells in lung cancer for years, and have illustrated the involvement of γδT17 cells in the effective immune surveillance of lung mucosa shaped by microbiota, as well as in the control of melanoma in the elderly (35, 36). It is worth pondering whether commensal bacteria engage in the maintenance of esophageal homeostasis and the occurrence and progression of EC, and importantly, whether the roles of γδT cells are involved. Recently, we also paid attention to the variations of tissue-resident γδT cells in surgical ESCC specimens, and hope to clarify the functions of these γδT cells in the tumor progression and its prognostic and diagnostic value in ESCC in future study.

## TAMs

Accounting for up to half of the total, TAMs are the most abundant infiltrated leukocyte in tumors and have two functionally polarized phenotypes in the TME: classically activated M1, and alternatively activated M2, macrophages (37). In the initial stages of various tumors, TAMs are preferentially polarized toward the M1 phenotype, producing abundant proinflammatory cytokines, including IL-12 and TNF, and exerting anti-tumor functions (38); however, on cancer progression and changes in the TME, TAMs, driven by tumor cell- and T cell-derived cytokines, including IL-4, IL-13, and IL-10, gradually acquire a polarized M2 phenotype, expressing mannose and the scavenger receptors, CD163 and CD204, and exhibit distinct functional properties that promote angiogenesis, as well as tissue remodeling and repair (37). The downstream signaling pathways activated by the numerous proteins and molecules produced by tumor cells and TAMs in the TME can increase TAM infiltration in EC, which is correlated with unfavorable prognosis and OS (39). For example, cysteine-rich angiogenic inducer 61 (Cyr61) from tumor cells and TAMs may contribute to the increase in CD204^+^ TAMs *via* MEK/ERK pathway activation in ESCC TME (40). Cancer cell-derived fibroblast growth factor 2 (FGF2) can facilitate TAM survival and migration through AKT/ERK signaling, activated by neural cell adhesion molecule 1-enhanced classical FGF receptor 1 (FGFR1) and intracellular FGF2/FGFR1 signaling. These tumor-infiltrating TAM are skewed toward CD163^+^ M2 phenotype under the action of the transcription factor, GATA3, and cytokines, including IL-4, IL-6, and IL-13, and promoted an immunosuppressive TME in EAC (33). The similar event happened in ESCC, for that CD68^+^PD-1^+^ TAMs in the ESCC TME are skewed toward an M2 phenotype (41), which can lead to elevation of tumor cell PD-L1 expression and promote tumor cell invasion and migration, associating with poor OS (42). Moreover, a study of EC patients who received neoadjuvant chemotherapy (NAC) followed by surgery demonstrated that high tumor CD163^+^ M2 macrophage infiltration is an independent predictor of response to NAC, and associated with poor prognosis and OS (43).

In fact, TAMs are involved in a variety of pathways that promote tumor progression of EC. The activation of the AKT/ERK pathway is the driving force to promote tumor cell growth, migration and invasion in EC (39, 44). This AKT/ERK pathway can be triggered by multiple factors derived from TAMs or cancer cells themselves, involving the FGF2/FGFR1 signaling we mentioned above (39), growth differentiation factor 15 induced in TAMs and derived from cancer cells (possibly through TGF-β type II receptor) (39, 44), overexpression of ANXA10 by cancer cells interacting with CD204^+^ TAMs (41), and high CXCL8 expression in TAMs and cancer cells (through the CXCL8-CXCR1/CXCR2 axis) (42), which are closely correlated with tumor invasion depth, lymph node metastasis and poor prognosis and OS of ESCC patients.

Another mechanism that the increased CD163^+^ TAM in the TME promote ESCC tumor progression is that they can augment angiogenesis by releasing thymidine phosphorylase (TP) under the influence of macrophage chemotactic protein-1(MCP-1) (45, 46), inducing vascular endothelial growth factor (VEGF) expression in EC cells (45, 47), and promoting stromal cell matrix metalloproteinase 9 (MMP9) production (48). CD163^+^ TAM distribution in tumor sites is closely related to EMT (49), possibly because their IL-1β production can enhance EMT, promoting tumor cell migration and invasion (50). Additionally, MCP-1 expression levels in the TME are positively correlated with increased stromal cell and TAM CC chemokine receptor 2 (CCR2) expression, associated with tumor invasion depth, lymph node metastasis, and distant metastasis, and predict poor prognosis in patients with ESCC (45–47).

**Figure 2:**
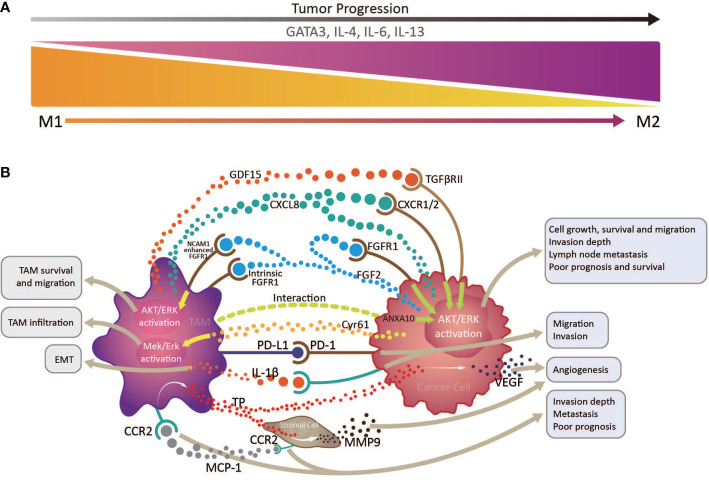
Interactions between tumor infiltrating macrophages, cancer cells, and stromal cells *via* multiple immune factors in the esophageal cancer tumor microenvironment. **(A)** tumor infiltrating macrophages (TAMs) transform from an anti-tumor M1 phenotype to a pro-tumor M2 phenotype with tumor progression, under the influence of the transcription factor, GATA3, and cytokines, including IL-4, IL-6, and IL-13. **(B)** By producing various immune factors, or interacting with cancer cells and stromal cells to promote their release of associated components, TAMs are closely correlated with epithelial to mesenchymal transition, angiogenesis, cancer cell survival and migration, invasion depth, and lymph node metastasis, and generally predict poor prognosis and survival in patients with esophageal cancer.

The majority of research on TAMs in EC has concentrated on ESCC. The clearly increased numbers of TAMs in both tumor structures and stroma is significantly negatively correlated with EC patient survival (43, 51–53). Under the combined action of various factors in esophageal TME, TAMs are gradually skewed toward an M2 phenotype, which is closely associated with angiogenesis and tumor aggressiveness, and thus predicts poor prognosis in patients with EC (**Figure 2**) (48). The M2/M1 macrophage ratio in esophageal tumors can serve as a sensitive indicator predicting lymph node metastasis and patient prognosis (54). To our knowledge, there is currently no TAMs-based immunotherapy strategy in EC. Study of an N-nitrosomethylbenzy-lamine-induced ESCC animal model suggested that CCL2-CCR2 signaling activation participates in TAM recruitment into the TME, which can promote immune evasion and tumor progression through the PD-1/PD-L2 pathway, indicating potential intervention and immunotherapy strategies targeting TAMs in patients with ESCC (47).

## DCs

As the main professional APCs, DCs are essential for triggering and regulating antigen-specific immune responses, and closely connect innate and acquired immunity. Human DCs are a heterogeneous population consisting of two types of conventional DC (cDC), cDC1 and cDC2, and plasmacytoid dendritic cell subsets in equilibrium, plus inflammatory DCs, which are generated in response to inflammation, and Langerhans cells (LCs), which originate from embryonic monocytes and can self-renew (55). DCs distributed in the esophageal mucosa are generally LCs, which remain in an immature immune state under normal conditions, rapidly maturing into professional APCs after capturing pathogen or tumor associated-antigens, to trigger T cell activation and immune responses (56). Mature DCs express various important markers, including CD80, CD86, and CD208. DCs at different stages of maturity are uniquely distributed in the ESCC TME. Abundant CD1α^+^ immature DCs are distributed in the cancerous epithelium, while fewer CD208^+^ mature DCs are present in the tumor stroma, particularly the peri-tumoral region (57). In ESCC, DC density indicates the immune defense status of the host against the carcinoma, as patients with marked DC infiltration in tumors survive for longer than those with low DC density (58).

DC maturity in the TME is closely related to cancer progression (59). Importantly, the amount of intratumor mature DCs expressing lysosome-associated membrane glycoprotein 3 (LAMP3), is closely associated with tumor-infiltrated CD8^+^ T cell numbers, which predict favorable prognosis in patients with ESCC (60). In clinical studies, preoperative chemoradiotherapy was shown to lead to significant increases high-mobility group box 1 (HMGB1) protein levels in the ESCC TME. HMGB1 is closely related to DC maturation and positively correlated with patient survival (61). Additionally, by stimulating cancer cells to release inflammatory chemokines (CCL2 or CCL20), IL-17A-producing cells can enhance CD1a^+^ DC infiltration of the TME, which is correlated with favorable OS of patients with ESCC (18). These reports highlight the connections between DC maturity and EC tumorigenesis and development.

Interactions of tumor and immune cells with the cytokines/chemokines they produce in the EC TME generate complex regulatory networks, which significantly impact DC phenotype and function, thereby influencing tumor progression. DC immune functions are impaired both in the circulation and at tumor sites in patients with EC, and this is accompanied by decreased CD80 and CD86 expression (62). The reduced activity and function of these infiltrated DCs involves mutated p53 protein overexpression in tumors (63). Compared with benign Barrett’s esophagus (BE), DC density is dramatically increased in adenocarcinoma (64), along with decreased C1q expression, which contributes to immune complex capture and subsequent classical complement activation pathway initiation, indicating the potential roles of DCs in EAC dysplasia and tumorigenesis (65). Esophageal tumor cells can also induce production of the tryptophan-catabolizing enzyme, indoleamine 2,3-dioxygenase 1 (IDO1) and/or expression of PD-L1 by immunosuppressive DCs, which can promote immune tolerance by inhibiting CD8^+^ T cell infiltration and inducing immunosuppressive Tregs, and are associated with poor prognosis in EC patients (3, 66–68). The density of receptor-binding cancer antigen expression on SiSo cells (RCAS1) in esophageal tumor tissues with dramatic DC infiltration was inclined to accompany a decrease in TILs, suggesting that RCAS1 can promote tumor cell escape from immune surveillance by inducing DC-activated TIL apoptosis (69). Further, CD47, a cell transmembrane protein expressed in ESCC tumor cells, can inhibit CD8^+^ T-cell infiltration and anti-tumor immune responses in a DC-dependent manner, by interacting with signal regulatory protein-α (SIRPα), expressed in DCs (70).

## MDSCs

MDSCs are generated in the bone marrow and rapidly differentiate into macrophages, DCs, neutrophils, eosinophils, basophils, and mast cells in healthy individuals; however, when cancer occurs, MDSCs can migrate into peripheral lymphoid tissues and tumor sites, contributing to TAM formation (71, 72). MDSCs can be polymorphonuclear (PMN-MDSCs) or mononuclear (M-MDSCs). PMN-MDSCs are morphologically and phenotypically similar to neutrophils, while M-MDSCs are similar to monocytes (71); both have immunosuppressive functions, mainly targeting T cells through Arginase-1 and inducible nitric oxide synthase-2 (iNOS) (73).

In ESCC patients, circulating MDSC numbers are elevated, accompanied by high PD-L1 expression (74). Concurrently, MDSC-derived TGF-β can induce high PD-1 expression on CD8^+^ T cells in the TME (75). Hence, MDSCs can exert immunosuppressive functions on T cells *via* the PD-1/PD-L1 pathway and are correlated with tumor burden, lymph node metastasis, and tumor stage (74). Importantly, IL-6 exerts vital roles in MDSC induction and their production of ROS, Arginase 1, and p-STAT3 (76). Circulating MDSC numbers and IL-6 levels in the TME are positively correlated with NLR, predicting poor OS in ESCC patients (77). The cell-cell junctions formed by interaction between p120ctn and E-cadherin are critical in maintaining normal esophageal epithelial homeostasis; however, p120ctn expression in the ESCC TME is decreased or absent, leading to E-cadherin degradation and NF-κB, AKT, and STAT3 phosphorylation in cancer cells, promoting cancer cell GM-CSF release, which can recruit MDSCs into the TME. NF-κB signaling activation in MDSCs up-regulates their IL-4RA expression and nitric oxide production, thereby inhibiting CD8^+^ T cell cytotoxicity and contributing to a TME conducive to tumor cell growth (78). Further, in a conditional p120-ctn knockout mouse model of oral-EC, expression of the receptor CD38 induced by tumor-derived IL-6, IGFBP-3, and CXCL16, promoted arrest of MDSC maturation in an immature state, with stronger inhibitory functions of activated T cells through production of iNOS, among other factors, thus promoting tumor growth (79).

## Neutrophils

Tumor-associated neutrophils (TANs) have a different phenotype and cell/chemokine activity from circulating neutrophils (80). TANs can be functionally divided into anti-tumor N1 and cancer-promoting N2 phenotypes. TGF-β in the TME contributes to the transformation of neutrophils from N1 to N2 (80, 81). Further, neutrophil phenotype and function in the TME change with tumor development. In early stage tumors, neutrophils are only on the tumor periphery and exhibit anti-tumor effects, while in later stages, they can penetrate the tumor and demonstrate pro-tumor effects (82). Studies on neutrophils in cancer have focused on the neutrophil to lymphocyte ratio (NLR), as it is impossible to classify N1/N2 neutrophils using surface markers (83). The NLR is usually derived from routine blood tests, and may reflect changes in the TME and systemic inflammation status (84), which are independent prognostic indicators in patients with EC (85). NLR and platelet-to-lymphocyte ratio are associated with EC progression (86), and elevated preoperative NLR is related to lymph node metastasis, deeper tumor invasion, and advanced TNM stage (87), predicting poor prognosis and OS in patients who have undergone esophagectomy (85, 88, 89). In addition to defending against microbial invasion through phagocytosis and degranulation, neutrophils can undergo apoptosis after activation, and then form neutrophil extracellular traps (NETs), fibrinoid structures comprising extracellular chromatin and granulocyte proteins, including myeloperoxidase and neutrophil elastase, which were discovered because of their pathogen-trapping function, which can promote tumor metastasis by capturing circulating tumor cells and causing their proliferation at a second site (83, 90). In EC patients without surgical stress or any other stimuli, tumors alone can induce high levels of circulating NETs, which are predictive of positive lymph node status, distant metastasis, and advanced disease stage (91).

The functions of neutrophils in the TME, influenced by various cytokines and/or chemokines, are controversial (92). By changing the esophageal microenvironment and gut bacteria, a high-fat diet can cause esophageal dysplasia, which promotes the development of BE into EAC, which involves IL-8 chemokine family activation and neutrophil recruitment, along with NK cell reduction, suggesting that increased neutrophils may inhibit NK cell-mediated tumor cell cytotoxicity and indicate poor prognosis (93). Conversely, in the ESCC TME, IL-17 [mainly produced by CD4^+^ Foxp3^-^ Th17 cells (17)] stimulates tumor cells chemokine (CXCL2 and CXCL3) production, causing accumulation and activation of myeloperoxidase^+^ TANs, which increase their killing capacity by releasing cytotoxic molecules, including IFN-γ, reactive oxygen species (ROS), and TNF-related apoptosis-inducing ligand, and predict favorable prognosis in ESCC patients (94).

## MCs and Eosinophils

MCs mainly localize to areas where organisms are likely to come into contact with pathogens or harmful substances, including the gastrointestinal tract, respiratory mucosa, and skin, and act as multifunctional immune cells involved in both innate and adaptive immunity in health and various disease states (95). In several human cancers, MCs recruited by stem cell factor or other mast cell activators in the TME, release angiogenic factors and proteases to promote blood vessel formation and degrade the extracellular matrix, leading to tumor cell invasion; however, they can also release ROS/TNF-α, tryptase, heparin, IL-1, IL-4, and IL-6, among other factors, to inhibit tumor cell growth and apoptosis (96). In the ESCC TME, high MC density is positively correlated with tumor angiogenesis, lymph node metastasis, invasion depth, and tumor progression (97, 98), and a predictor of poor survival in ESCC patients (97, 99). Furthermore, activated MCs in EC tissue express high levels of tissue kallikrein (TK1), which may subsequently generate mitogenic kinin, a promoter of tumor cell growth (100). Interestingly, another study found that the presence of a group of MCs able to produce IL-17 in the esophageal muscularis propria, rather than in tumor nests, is positively correlated with the level of activated CD169^+^ macrophages and effector CD8^+^ T cells in the same region, indicating favorable prognosis and survival (101). It is noteworthy that IL-17 released from Th17 cells in the EC TME, as we mentioned above, was involved in recruiting cells with anti-tumor effects including NK cells, neutrophils, and CD1α^+^ DCs, so whether MCs participated in this process deserves further attention.

Eosinophils usually cluster together with MCs in tissue sites under both homeostatic and inflammatory conditions (102). With similar developmental and functional patterns, such as releasing cationic proteins pre-stored in cytoplasmic granules by degranulation upon activation, they often participate in host responses to helminth infection and allergic disease in a synergistic manner (102, 103). Based on their abilities to release cytokines, eosinophils are being recognized to be also involved in local immunity, tissue homeostasis, remodeling, and repair in multiple previously unexpected tissues, especially the mucosal tissues such as gut and esophageal (104–106). For example, eosinophil infiltration is a typical feature of eosinophilic esophagitis, an allergic disease associated with epithelial barrier dysfunction and chronic type 2 inflammation (106). Other than this, the increase of eosinophils is also found in some ESCC patients (107), positively correlating with low incidence of LN metastasis in patients with early ESCC and predicting favorable OS in ESCC patients treated with concurrent chemoradiotherapy (108, 109). Conversely, eosinophils appear to be significantly reduced across all stages of dysplasia and EAC progression, indicating the loss of immune surveillance by eosinophils may contribute to BE progression toward dysplasia and cancer (110). Indeed, eosinophils play controversial roles in modulating tumor initiation and progression, for that they are both the source of anti-tumorigenic factors including TNF-a, granzyme, cationic proteins, and IL-18, and protumorigenic molecules such as pro-angiogenic factors, depending on the different immune milieu (35). Nevertheless, how eosinophils exert their functions in the occurrence and development of EC remains unclear. There is still a long way to go to understand the specific mechanism of eosinophils in EC, which will shed light on the control of EC progression and the immunotherapy based on them.

## Crosstalk and Regulation of Innate Immune Cells

**Figure 3:**
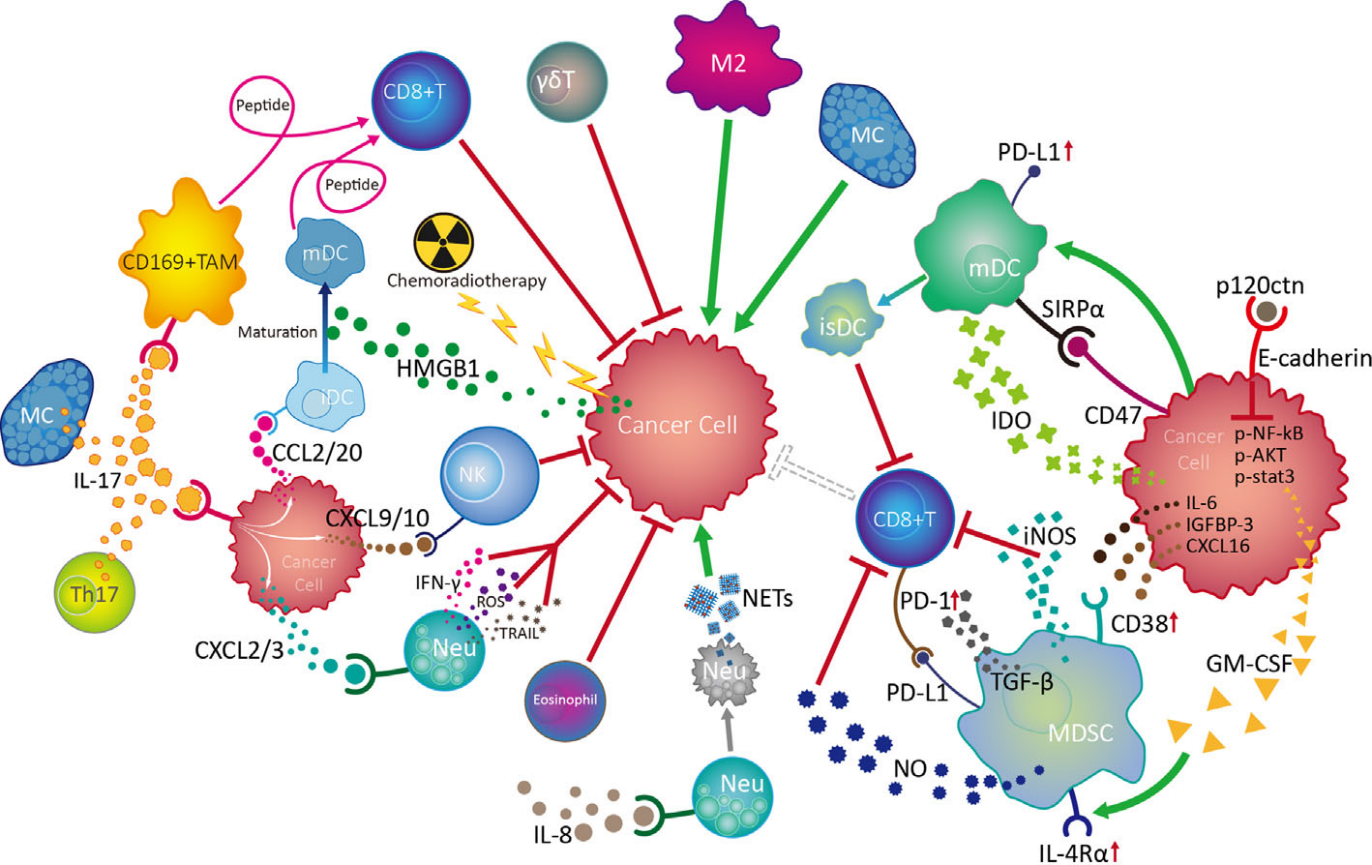
Crosstalk and regulation of innate immune cells in the esophageal cancer tumor microenvironment. Among the multiple innate immune cells involved in esophageal cancer progression, NK and γδT cells are active in the front line of anti-tumor defences with their powerful cytotoxicity. Mature dendritic cells (mDC) play vital roles in antitumour responses by boosting the function of CD8+ effector T cells, while chemoradiotherapy can promote DC maturity by increasing HMGB1 levels in the tumor microenvironment (TME). IL-17 derived from Th17 cells and MCs can activate CD169+ tumor infiltrating macrophages (TAMs) and effector CD8+ T cells, as well as recruit NK cells, CD1a+ immature DCs (iDC) and neutrophils (Neu) into the TME by stimulating cancer cells to release various chemokines, thereby exerting antitumour effects. Although the mechanism is unknown, eosinophils are also involved in the anti-tumor process. On the contrary, neutrophils can also promote tumor development by inhibiting NK cell function in response to IL-8, as well as by forming NETs after tumor-induced apoptosis. MCs can promote tumor cell growth through the TK1/mitogenic kinin pathway. Importantly, tumor cells can escape from innate immune surveillance by promoting TAM progression to a suppressive M2 phenotype, which inhibits CD8^+^ T cell function by transforming DCs into an immunosuppressive phenotype (isDC) and recruiting myeloid-derived suppressor cells to inhibit the cytotoxic effects of CD8^+^ T and NK cells.

The EC TME contains a various innate immune cells and associated cytokines/chemokines. By regulating or being regulated, innate immune cells and diverse other cell populations, including adaptive immune cells, stromal cells, and cancer cells, form complex regulatory networks through receptor-ligand binding and immune factor release in the TME, which influences the proliferation, migration, and invasion of cancer cells, as well as angiogenesis, thus influencing tumor growth and metastasis (**Figure 3**).

The balance of activating and inhibitory receptor expression, cytokine release, and degranulation ability endow NK cells with powerful immune surveillance and direct killing functions toward tumor cells; however, NK cells exhibit a dysfunctional phenotype and exhaustion on tumor development of EC, involving cancer cell-induced over-expression of inhibitory receptors, such as PD-1, and suppression of activated receptors, such as NKp30, tumor infiltrating macrophage-induced CD56^dim^ NK cell apoptosis *via* H_2_O_2_, and aggregation of neutrophils under the influence of IL-8. As the most common infiltrating immune cells in the tumor milieu, TAMs are polarized into an inflammatory M1 phenotype during early EC, facilitating CD8^+^ T cell activation and exerting anti-tumor effects. As EC develops, TAMs gradually transform into an M2 phenotype, promoting tumor cell proliferation, migration, and invasion. DC maturation in the TME can be promoted by HMGB1 stimulated by preoperative chemoradiotherapy and is accompanied by surface molecule (CD80, CD86, CD208, and LAMP3) expression with anti-tumor functions of tumor-associated antigen presentation, which facilitates effective CD8^+^ T cell activation. Nevertheless, DC activity and function can be damaged by cancer cells overexpressing mutated p53. EC tumor cells can also escape from immune surveillance by inducing DC-triggered TIL apoptosis under the influence of RCAS1, or by promoting the DC transformation into an immunosuppressive phenotype, with PD-L1 and SIRPα expression and IDO1 production, inhibiting CD8^+^ T cell activity. Moreover, tumors can induce high levels of circulating NETs from apoptotic neutrophils, which predict positive lymph node status, distant metastasis, and advanced stage in EC. Additionally, MDSCs in EC TME can be recruited and activated by cancer cell-derived cytokines/chemokines (IL-6, IGFBP-3, CXCL16, and GM-CSF), thereby accelerating tumor progression by inhibiting CD8^+^ T cell activation. Controversially, MCs in EC can both promote tumor cell growth through TK1/mitogenic kinin signaling and exert anti-tumor effects, through activation of CD169^+^ TAMs and CD8^+^ effector cells. Importantly, IL-17 from Th17 cells and MCs in EC TME can promote cancer cell release of CXCL9/10, CXCL2/3, and CCL2/20, which can increase the infiltration and anti-tumor effects of NK cells, neutrophils, and CD1α^+^ DCs, respectively, and predict favorable prognosis for patients with esophageal tumors.

## Immunotherapy Strategy Based on Innate Immune Cells

Treatment for EC remains less than satisfactory. Recent studies indicate that treatment with a single PD-1 inhibition agent is more effective for ESCC than EAC, while a combination of inhibitors targeting PD-1 with chemotherapy is a good strategy for treatment of metastatic disease (68); however, partly due to a lack of reliable predictive indicators of whether patients respond effectively, the use PD-1 inhibitors, alone or with other checkpoint antibodies, has had controversial results (36). Problems remain for use of PD-L1 as a predictive biomarker, because of tumor heterogeneity, a lack of reproducibility of results, and a complex scoring system (68). Therefore, it is imperative to identify new predictive indicators and immunotherapy strategies.

**Figure 4:**
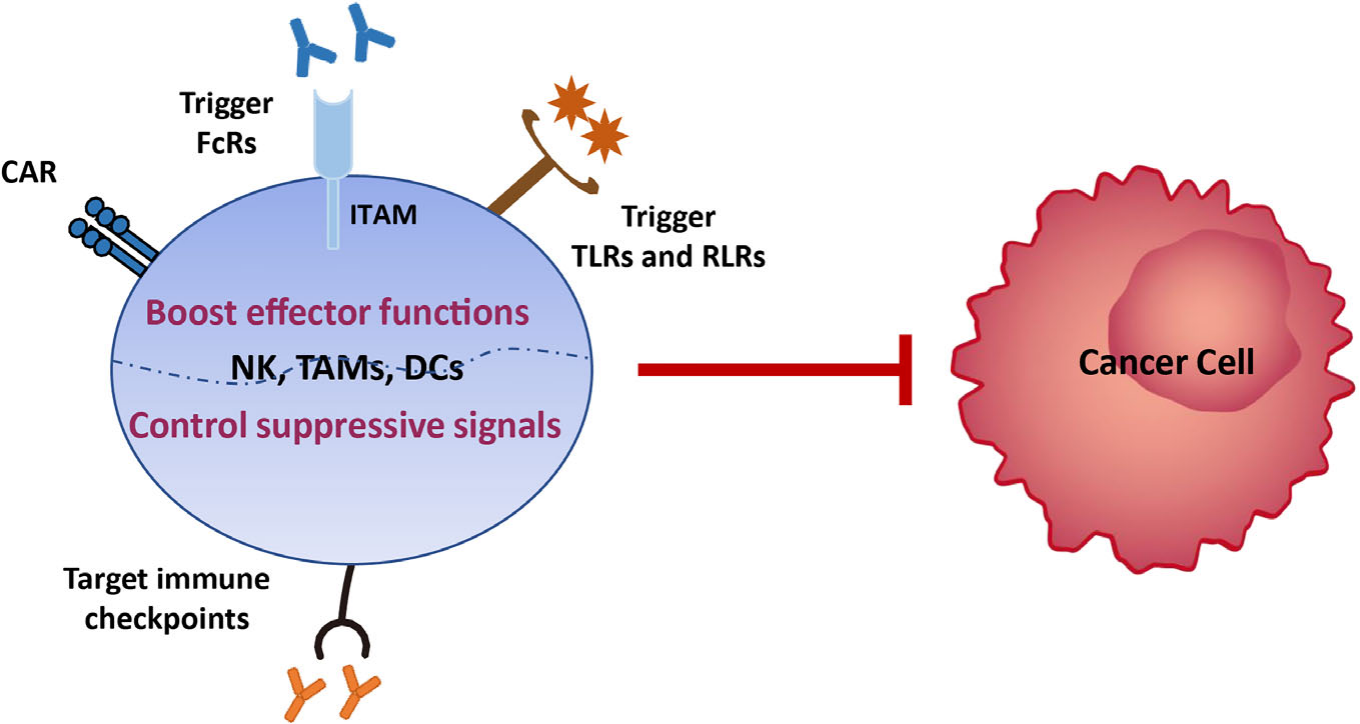
Immunotherapy strategies based on innate immune cells. Various tumor treatment regimens can be implemented by boosting the effector functions of innate immune cells, including generating engineered CAR NK cells with high anti-tumor activity and CAR macrophages which can be polarized towards an anti-tumor M1 phenotype, developing anti-tumor antibodies that can bind to activating FcRs expressed on innate immune cells lacking antigen receptors, and enabling them to act specifically on EC cells, and targeting toll-like receptors (TLRs) and RIG-I-like receptors (RLRs), to generate a ‘pathogen-induced-like’ innate immune responses at the tumor site, since the innate immune system can sense the nucleic acids of growing tumors using the pathogen and damage receptors involved in infection detection. Another immunotherapy strategy is to control immunosuppression signals on innate immune cells *via* development of broad-spectrum immune checkpoint inhibitors targeting NK cells (i.e., LAG3, TIM3, and PD-1, which we review here, and NKG2A, CTLA4, TIGIT, and CD96 which require further investigation) and/or myeloid cells (i.e., SIRPα).

Various tumor treatment regimens aim to enhance effector cell function and/or control immunosuppression (9). Hence, there is potential to treat EC by boosting innate immune functions, such as NK cell cytotoxicity, phagocytosis, and DC maturation, which subsequently activate and sustain tumor-specific CD8^+^ T cell effects. First, the development of broad-spectrum immune checkpoint inhibitors targeting NK cells (i.e., LAG3, TIM3, and PD-1, which we review here, and NKG2A, CTLA4, TIGIT, and CD96 which require further investigation) and/or myeloid cells (i.e., SIRPα), is a promising approach that should be advanced in EC therapy. NK cells share the majority of checkpoints with T cells; therefore, inhibition of these receptors will also release various brakes on T cells and benefit both innate immunity and T-cell functions. Second, the development of anti-tumor antibodies that can bind to activating FcRs expressed on innate immune cells lacking antigen receptors, such as NK cells and macrophages, will enable them to act specifically on EC cells. Third, multiple pattern recognition receptors expressed on the surface of innate immune cells in mucosal sites ensure rapid responses to pathogenic microorganisms by recognizing pathogen-related molecular patterns. Therefore, targeting toll-like receptors (TLRs), RIG-I-like receptors (RLRs), and stimulators of interferon, to generate a ‘pathogen-induced-like’ innate immune responses at the tumor site, may be promising approaches in EC treatment, since the innate immune system can sense the nucleic acids of growing tumors using the pathogen and damage receptors involved in infection detection. Finally, generating engineered CAR NK cells with high anti-tumor activity and CAR macrophages which can be polarized towards an anti-tumor M1 phenotype, in addition to, or instead of, CAR T cells, may provide a route to next generation immunotherapies for EC (**Figure 4**).

In conclusion, the application of these methods to clinical treatment is based on sufficient research of innate immune functions in the EC TME and numerous preclinical trials. Indeed, combined therapy approaches may become the norm in future treatment of EC.

## Perspective

The roles of innate immune cells in mucosal tissues in maintaining regional homeostasis and in host resistance to infection and tumor has been extensively elaborated. However, partly due to specimen constraints and regional disparities in incidence (i.e., its higher prevalence in developing countries, particularly East Asia) (2), few studies have focused on innate immunity in EC, and ongoing immunotherapy of patients with esophageal tumors is almost entirely restricted to targeting of the PD1/PD-L1 pathway (68). Based on the limited available data, one limitation of this review is that we cannot comprehensively compare the similarity or difference of innate immune cells between ESCC and EAC.

Although previous research provides clues to the essential roles of various innate immune cells in the EC TME, considerable further investigations of their functions in EC occurrence and development are required. It is worth noting that the inadequacy and imbalance of the previous studies may lead to incomplete evaluation of the complicated immune contexture of EC. For example, γδT and NKT cells, two typical innate immune cells which are deeply involved in anti-tumor responses to multiple cancers, have rarely been studied in EC. Recently, a group of innate lymphocytes, innate lymphoid cells (ILCs), were identified. ILCs can be subdivided into ILC1, ILC2, and ILC3, subtypes, based on cytokine production and transcription factors associated with their development (111). Alternatively, ILCs can be classified as cytotoxic (i.e., conventional NK cells) and helper ILCs, which resemble the T cell classification (i.e., CD8^+^ cytotoxic T and CD4^+^ T helper cells) (112). In addition to circulating cytotoxic NK cells, ILCs exhibit clear tissue tropism, preferentially localizing to barrier tissues, including the lung, intestine, and skin, and involving in inflammation and carcinogenesis (112). Whether ILCs in the esophageal mucosa participate in the development of esophageal diseases, such as BE and EC remains unknown; hence, the functions of these cells in EC warrants attention in future preclinical and clinical studies. Overall, a more comprehensive understanding of the roles of innate immune cell populations in EC and identification of better treatment targets will likely ultimately benefit patients with EC.

## Author Contributions

KC designed and drafted the manuscript. SH participated in writing the manuscript. XM provided and contributed fruitful discussion. MC started the study and participated in the paper writing. All authors contributed to the article and approved the submitted version.

## Funding

This work was supported by Natural Science Foundation of Anhui Province (2008085MH277), National Natural Science Foundation of China (81471552), the Anhui Provincial Project of the Key Laboratory of Tumor Immunotherapy and Nutrition Therapy (2019b12030026).

## Conflict of Interest

The authors declare that the research was conducted in the absence of any commercial or financial relationships that could be construed as a potential conflict of interest.

## Abbreviations

APCs, antigen-presenting cells; BE, Barrett’s esophagus; CAR, chimeric antigen receptor; CCR2, CC chemokine receptor 2; DCs, dendritic cells; EC, esophageal cancer; EAC, esophageal adenocarcinoma; ESCC, esophageal squamous cell carcinoma; FcR, Fc receptor; FGF2, fibroblast growth factor 2; FGFR1, fibroblast growth factor receptor 1; HMGB1, high-mobility group box 1; HSP, heat shock protein; IDO1, indoleamine 2,3-dioxygenase 1; iNOS, inducible nitric oxide synthase-2; ILCs, innate lymphoid cells; LAMP3, lysosome-associated membrane glycoprotein 3; MCs, mast cells; MCP-1, macrophage chemotactic protein-1; MDSCs, myeloid-derived suppressor cells; M-MDSCs, mononuclear myeloid-derived suppressor cells; MMP9, matrix metalloproteinase 9; NETs, neutrophil extracellular traps; NK, natural killer; NAC, neoadjuvant chemotherapy; NLR, neutrophil to lymphocyte ratio; OS, overall survival; PMN-MDSCs, polymorphonuclear myeloid-derived suppressor cells; RCAS1, receptor-binding cancer antigen expression on SiSo cells; RLRs, RIG-I-like receptors; SIRPα, signal regulatory protein-α; TLRs, targeting toll-like receptors; Tregs, regulatory T cells; TK1, tissue kallikrein; TAMs, tumor-associated macrophages; TANs, tumor-associated neutrophils; TME, tumor microenvironment; TNF, tumor necrosis factor; TP, thymidine phosphorylase; VEGF, vascular endothelial growth factor.
