# Corrigendum: Innate Immune Cells in the Esophageal Tumor Microenvironment

**DOI:** 10.3389/fimmu.2021.708705

**Published:** 2021-05-27

**Authors:** Kele Cui, Shouxin Hu, Xinyu Mei, Min Cheng

**Affiliations:** ^1^ Department of Clinical Laboratory, The First Affiliated Hospital of USTC, Division of Life Sciences and Medicine, University of Science and Technology of China, Hefei, China; ^2^ Department of Geriatrics, Gerontology Institute of Anhui Province, The First Affiliated Hospital of USTC, Division of Life Sciences and Medicine, University of Science and Technology of China, Hefei, China; ^3^ Anhui Provincial Key Laboratory of Tumor Immunotherapy and Nutrition Therapy, Hefei, China; ^4^ Cancer Immunotherapy Center, The First Affiliated Hospital of USTC, Division of Life Sciences and Medicine, University of Science and Technology of China, Hefei, China; ^5^ Department of Thoracic Surgery, The First Affiliated Hospital of USTC, Division of Life Sciences and Medicine, University of Science and Technology of China, Hefei, China

**Keywords:** innate immune cells, crosstalk, regulation, esophageal tumor microenvironment, immunotherapy strategy

In the published article, there was an error in affiliations 1, 2, 4 and 5. Instead of “^1^ Department of Clinical Laboratory, Division of Life Sciences and Medicine, The First Affiliated Hospital of USTC, University of Science and Technology of China, Hefei, China; ^2^ Department of Geriatrics, Gerontology Institute of Anhui Province, Division of Life Sciences and Medicine, The First Affiliated Hospital of USTC, University of Science and Technology of China, Hefei, China; ^4^ Cancer Immunotherapy Center, Division of Life Sciences and Medicine, The First Affiliated Hospital of USTC, University of Science and Technology of China, Hefei, China; ^5^ Department of Thoracic Surgery, Division of Life Sciences and Medicine, The First Affiliated Hospital of USTC, University of Science and Technology of China, Hefei, China”, it should be “^1^ Department of Clinical Laboratory, the First Affiliated Hospital of USTC, Division of Life Sciences and Medicine, University of Science and Technology of China, Hefei, China; ^2^ Department of Geriatrics, Gerontology Institute of Anhui Province, the First Affiliated Hospital of USTC, Division of Life Sciences and Medicine, University of Science and Technology of China, Hefei, China; ^4^ Cancer Immunotherapy Center, the First Affiliated Hospital of USTC, Division of Life Sciences and Medicine, University of Science and Technology of China, Hefei, China; ^5^ Department of Thoracic Surgery, the First Affiliated Hospital of USTC, Division of Life Sciences and Medicine, University of Science and Technology of China, Hefei, China”.

The authors apologize for this error and state that this does not change the scientific conclusions of the article in any way. The original article has been updated.

